# High Anti-Swelling Zwitterion-Based Hydrogel with Merit Stretchability and Conductivity for Motion Detection and Information Transmission

**DOI:** 10.3390/nano15131027

**Published:** 2025-07-02

**Authors:** Qingyun Zheng, Jingyuan Liu, Rongrong Chen, Qi Liu, Jing Yu, Jiahui Zhu, Peili Liu

**Affiliations:** Key Laboratory of Superlight Materials and Surface Technology, Ministry of Education, College of Materials Science and Chemical Engineering, Harbin Engineering University, Harbin 150001, China; zqy1233163@163.com (Q.Z.); chenrongrong@hrbeu.edu.cn (R.C.); qiliu@hrbeu.edu.cn (Q.L.); jing.yu@hrbeu.edu.cn (J.Y.); jiahuizhu@hrbeu.edu.cn (J.Z.); liupeili@hrbeu.edu.cn (P.L.)

**Keywords:** zwitterion, hydrogel, anti-swelling property, wearable sensor

## Abstract

Hydrogel sensors show unique advantages in underwater detection, ocean monitoring, and human–computer interaction because of their excellent flexibility, biocompatibility, high sensitivity, and environmental adaptability. However, due to the water environment, hydrogels will dissolve to a certain extent, resulting in insufficient mechanical strength, poor long-term stability, and signal interference. In this paper, a double-network structure was constructed by polyvinyl alcohol (PVA) and poly([2-(methacryloyloxy) ethyl]7 dimethyl-(3-sulfopropyl) ammonium hydroxide) (PSBMA). The resultant PVA/PSBMA-PA hydrogel demonstrated notable swelling resistance, a property attributable to the incorporation of non-covalent interactions (electrostatic interactions and hydrogen bonding) through the addition of phytic acid (PA). The hydrogel exhibited high stretchability (maximum tensile strength up to 304 kPa), high conductivity (5.8 mS/cm), and anti-swelling (only 1.8% swelling occurred after 14 days of immersion in artificial seawater). Assembled as a sensor, it exhibited high strain sensitivity (0.77), a low detection limit (1%), and stable electrical properties after multiple tensile cycles. The utilization of PVA/PSBMA-PA hydrogel as a wearable sensor shows promise for detecting human joint movements, including those of the fingers, wrists, elbows, and knees. Due to the excellent resistance to swelling, the PVA/PSBMA-PA-based sensors are also suitable for underwater applications, enabling the detection of underwater mannequin motion. This study proposes an uncomplicated and pragmatic methodology for producing hydrogel sensors suitable for use within subaquatic environments, thereby concomitantly broadening the scope of applications for wearable electronic devices.

## 1. Introduction

A substantial body of research has emerged on the potential of underwater hydrogel sensors in various fields. These sensors have exhibited notable promise for applications in diverse areas [1,2,3,4,5], including medical health monitoring [6], sports performance monitoring [7], and human–machine interaction [8]. The rationale behind this phenomenon is attributable to the materials’ unparalleled properties, including flexibility, biocompatibility, high sensitivity, and tunable physical and chemical properties [9,10,11,12]. The potential applications of underwater hydrogel sensors are vast and include areas such as underwater exploration [13], environmental monitoring [14], marine biological research [7,15], and military applications. The advent of smart materials and soft robotics has precipitated the exploration of novel applications for underwater hydrogel sensors in domains such as ocean exploration, environmental monitoring, and military applications. Liu et al. developed a soft robot skin composed of two-dimensional materials (2DMs) and water-repellent soft robots that exhibit multifunctionality, including stretchability, temperature regulation, and resistance to deformation [16]. Ding et al. designed a soft robot skin that uses water-repellent sensors to detect water waves, demonstrating its potential for use in marine environments [17]. Despite the substantial progress achieved in the field of underwater hydrogel sensors, there is considerable scope for enhancement in the mechanical strength, environmental stability, and signal response speed.

Due to their high hydrophilicity, water-based materials inevitably undergo swelling upon exposure to water, leading to structural failure, functional deterioration, and layer separation [18,19]. To address this issue, a new class of materials, known as “water-repellent water-based materials”, has emerged. These materials employ sophisticated strategies, such as chemical crosslinking [20], surface modification [21], and nanocomposite [22] formation to enhance their environmental stability and long-term reliability. Wang et al. proposed a novel method for fabricating water-repellent composite materials (SGC) by employing in situ polymerization to integrate a conductive water-repellent material (MXene) with a lipid-based composite (Lipogel), thereby imparting water-repellent properties to the resulting composite [23]. Qi et al. investigated the interaction between CTAB and P, which is a constituent of polyacrylate [24]. In the domain of material science, the electrostatic repulsion and the effect of water repulsion due to the presence of a certain compound have been shown to result in the prevention of dissolution. Typically, a double-network structure (DNS) water-repellent gel is composed of two distinct network materials, exhibiting high strength and durability [25,26,27]. The fundamental structural composition of the gel consists of two networks. One of them is rigid and the other is fragile, while the second is flexible and extensible. The rigidity of the first network dissipates the tensile stresses present in the water-repellent gel, while the flexible and less-intertwined second network provides support to the gel, maintaining its shape [28]. Du et al. utilized polyacrylamide as the backbone network and Al^3+^ complex structure as the secondary network to prepare double-mesh hydrogels, which exhibited superior mechanical recovery and mechanical properties [29]. This dual network configuration endows the water-repellent gel with exceptional mechanical properties, e.g., Dou et al. demonstrated that their composite hydrogel consists of chitosan (CS) and poly (N-acryloyl 2-glycine) (PACG). The resulting composite hydrogel readily formed a shell-structured DNA hydrogel, exhibiting excellent anti-swelling properties [30]. Additionally, the interpenetration of the two networks increases the number of points of contact between the polymer chains, thereby reducing the porosity of the gel. Consequently, double-network water-repellent gels generally exhibit lower dissolution behavior. However, the high density and interconnectedness of the rigid network can impede the movement of water molecules, thereby restricting the movement of the water-repellent gel. Consequently, the development of water-repellent gels that exhibit both high electrical properties and mechanical strength remains an endeavor.

Polyvinyl alcohol (PVA) has the advantages of good biocompatibility [31], non-toxicity [32], chemical stability [33], and low price, and is widely used in wearable flexible sensors [34]. Liu et al. chose to use the conductive polymer PEDOT blended with PVA to prepare hydrogels with good electrical conductivity, but the hydrogels in this work could not be applied underwater due to their inherent hydrophilicity [35]. In contrast to chemical and radiation crosslinking methods, physical crosslinking does not necessitate the incorporation of crosslinking agents. Instead, it relies on low-energy freezing and thawing cycles to partially crystallize the molecular chains in the aqueous PVA solution, thereby forming physical crosslinking points and conferring a degree of mechanical strength and stability to the hydrogel [36]. Cyclic freezing and thawing to produce a eutectic can greatly increase the crosslinking density of PVA hydrogel and thus improve its mechanical properties [37]. SBMA ([2-(methacryloyloxy) ethyl] dimethyl-(3-sulfopropyl) ammonium hydroxide) is composed of two distinct components: a positively charged sugar base and a negatively charged sulfonate base. It exhibits both optimal biocompatibility and high-affinity binding properties for protein-based non-specific adsorption [38]. The behavior of PSBMA is pH-dependent, remaining neutral at higher pH and becoming charged at lower pH. Consequently, during the construction of a dual network structure, an accumulation of internal static charges is observed, facilitating the expulsion of water molecules and thereby achieving an anti-solvent swelling effect [39]. The polymeric material contains six phosphoric acid groups (-PO_4_^3−^), which facilitate its interaction with both PVA’s hydroxyl groups (-OH) and PSBMA’s sulfonic acid groups (-SO_3_^−^). This interaction results in forming a high-density hydrogen bond, which suppresses the penetration of water molecules [40]. This process also results in an augmentation of the number of PVA crystals in the frozen state, a reduction in the rigidity of the water-based composite, and a subsequent attainment of a balance between flexibility and anti-solvent properties [41]. In this paper, PA-modified dual-network-structured PVA/PSBMA hydrogels (named as PVA/PSBMA-PA hydrogel) are utilized to achieve this objective. To determine the stability of a water-repellent PVA/PSBMA-PA in various conditions, the experiment involves the development of an electronic sensor that can detect movement and facial expressions in real time. Meanwhile, this sensor can be used to monitor signals transmitted underwater.

## 2. Materials and Experiment Section

### 2.1. Materials

Polyvinyl alcohol 1799 (PVA, Mn = 75,000, ≈98–99% alcoholysis), [2-(methacryloyloxy) ethyl] dimethyl-(3-sulfopropyl) ammonium hydroxide (SBMA, 99%), phytic acid (PA, 50 wt% in water), N, N, N′, N′-tetramethylethylethylenediamine (TEMED, 99%), and potassium persulfate (KPS, 99%) were purchased from Aladdin (Shanghai, China).

### 2.2. Preparation of PVA/PSBMA-PA Hydrogel

Initially, 2 g of PVA was dissolved in 14 mL of deionized water by stirring at 95 °C for two hours until complete dissolution was achieved, and then cooled to 55 °C. The mixture containing a specific quantity of SBMA and PA solution was added and thoroughly mixed. Then, the mixed solution was maintained at ambient temperature for three hours to facilitate the removal of bubbles. Subsequently, 50 μL of TEMED and 0.03 g of KPS were incorporated and agitated for 15 min to ensure uniform distribution. Then, the mixture was transferred into a glass plate membrane apparatus, which was then maintained at 60 °C for two hours. After the polymerization, the hydrogel was frozen at −20 °C for 16 h, then thawed at room temperature for 8 h and cycled twice. The prepared PVA/PSBMA-PA hydrogels with different PA additions were named PSP-1, PSP-2, PAP-3, and PSP-4. For comparison, PVA single-network hydrogel and PVA/PSBMA double-network hydrogel were synthesized.

## 3. Results and Discussion

### 3.1. Characterization of Hydrogel

**Figure 1:**
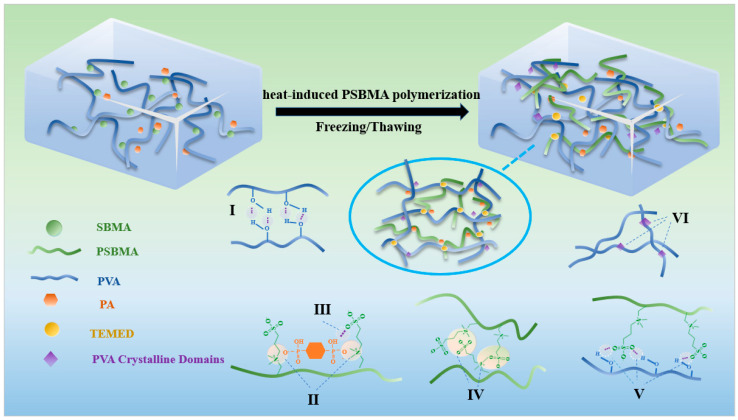
Schematic diagram and formation mechanism of PVA/PSBMA-PA hydrogel.

As shown in Figure 1, the hydrogel with a bilayer network structure consisted of flexible PVA and rigid PSBMA. Firstly, there was a partial hydrogen bonding (I) in the PVA solution. Subsequently, the mixture was introduced into a PVA solution to yield a double network, with the polymerization initiated by KPS and TEMED. After the polymerization of SBMA was achieved by thermal initiation, there was a hydrogen bonding (III) and electrostatic interaction (II) between PA and PSBMA. The -SO_3_^−^ groups in PSBMA were pH-controllable, meaning that they could be protonated to -SO_3_H and form hydrogen bonds within the hydrogel in an acidic environment. Concurrently, with a substantial number of hydrogen bonds, the PA could form electrostatic interactions with the positively charged quaternary ammonium (-R_3_N^+^) group in PSBMA. There was also an electrostatic interaction between PSBMA molecules (IV), and there was an electrostatic interaction between the -OH on the PVA and -SO_3_^−^ on the PSBMA (V). Finally, the PVA/PSBMA-PA hydrogel was fabricated expeditiously by subjecting the solution to repeated cycles of freeze and thaw, and molecular chains in the PVA were partially crystallized (VI). The presence of hydrogen bonds and electrostatic interactions within the hydrogel has been shown to reduce its swelling rate.

The elemental mapping of the PVA/PSBMA-PA hydrogel (see Figure S1) revealed an even distribution of the elements O, N, S, and P, indicating the successful formation of the hydrogel. The formation of the PVA/PSBMA-PA hydrogel was investigated through analysis of the FTIR spectra, SEM, and EDS spectra, presented in Figures S2 and S3. Compared with PVA hydrogel and PVA/PSBMA hydrogel, PVA/PSBMA-PA hydrogel had a more surface cavity structure. The peak of -OH broadened and shifted to a lower wavenumber (from the usual 3400 cm^−1^ to about 3200 cm^−1^). During the freezing/thawing process, more hydrogen bonds were formed between the chains of the PVA molecules, resulting in a restricted vibration of the -OH group and an increase in the width of the peaks [42]. The formation of the hydrogen bonding network also reduced the force constant of the O-H bonds, causing the absorption peaks to redshift. The freezing/thawing cycle promoted the orderly arrangement of PVA molecular chains [43] and increased the crystallinity, so the peak at 1140 cm^−1^ existed. Compared with pure SBMA, both PVA/PSBMA and PVA/PSBMA-PA hydrogels exhibited a P=O peak at 1050 cm^−1^. At the same time, the C=O absorption vibration peak shifted from 1725 cm^−1^ to 1722 cm^−1^ in the PVA/PSBMA hydrogel, and further to 1719 cm^−1^ in the PVA/PSBMA-PA hydrogel, indicating that the hydrogen bonds between the ester groups became more robust. The SO_3_^−^ stretching vibration peak at 1035 cm^−1^ shifted to 1039 cm^−1^ in the PVA/PSBMA hydrogel and 1043 cm^−1^ in the PVA/PSBMA-PA hydrogel, verifying the protonation of the -SO_3_H structure. Typically, the presence of hydrogen bonding within a molecule causes a red shift in the infrared characteristic peaks. However, in this hydrogel, the simultaneous presence of a strong electrostatic interaction led to a blueshift of the -OH peaks [44].

### 3.2. Mechanical Properties of PVA/PSBMA-PA Hydrogel

**Figure 2:**
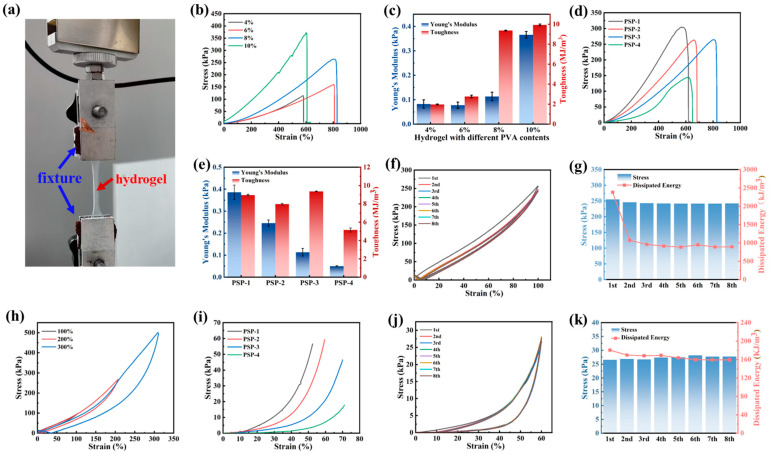
Mechanical properties of hydrogels: (**a**) Schematic tensile diagram of PVA/PSBMA-PA hydrogels. (**b**) Stress–strain curves and (**c**) toughness and Young’s modulus of hydrogels with different PVA contents. (**d**) Stress–strain curves and (**e**) toughness and Young’s modulus of hydrogels with different PA additions. (**f**) Tensile curves and (**g**) maximum tensile strength and dissipated energy of PVA/PSBMA-PA hydrogels with a morphology of 100% cycling for 8 cycles. (**h**) Tensile cyclic curves at different strains (100%, 200%%, and 300%). (**i**) Compression curves of PVA/PSBMA-PA hydrogels at different PA additions, (**j**) compression cyclic curves at 60% strain with 34% PA addition, and (**k**) maximum compression strength and hysteretic energy of the corresponding cycle.

Flexible hydrogel devices must demonstrate exceptional mechanical properties, particularly with regard to tensile and compressive strength, in order to be suitable for use during arbitrary limb movements and to prevent damage during actual applications [45,46]. The PVA/PSBMA-PA hydrogel exhibited no fracture when subjected to tensile testing (shown in Figure 2a), which exceeded approximately four times its length. According to Figure S5, the toughness of the hydrogels increased after the introduction of PSBMA chain segments, which was due to the energy consumption by sliding of PSBMA chain segments during tensile deformation. Compared to the PVA/PSBMA hydrogel, the toughness and ductility of the hydrogel increased dramatically with the addition of PA, which was due to the large amount of hydrogen bonding and electrostatic interactions generated within the hydrogel after the addition of PA. The first network of the hydrogel, the PVA network, increased in strength and modulus, with a gradual increase in the percentage of its content (Figure 2b,c), but its elongation decreased. This was due to the excessive content of PVA after freezing/thawing treatment, where the crosslinking density increased, and the micro-crystalline domains inside became more [47]. As illustrated in Figure 2d,e, the outcomes of the mechanical properties of the PVA/PSBMA-PA hydrogel following treatment with varying amounts of PA can be seen. An initial increase in stress, strain, and toughness was observed with increasing PA dosages. However, a subsequent decrease in PA dosages was accompanied by a gradual increase in elongation. As the percentage of PA exceeded 34%, a decline in elongation and toughness was observed. A divergent trend was observed for Young’s modulus. In contrast, as the dosage of PA increased, the modulus decreased. At very low PA content, electrostatic interactions between -R_3_N^+^ and -SO_3_^−^ groups in PSBMA resulted in the formation of a collapsed network of dense polymer chains. The introduction of these rigid chains increased Young’s modulus [48]. The incorporation of PA content at a rate of 34% led to the equilibrium of negative charges, thereby facilitating the formation of hydrogen bonds between -SO_3_H and polymers. This prevented the polymer chains from slipping in case of overstretching. The tensile strength was measured at 304 kPa, while the toughness was determined to be 0.39 MJ/m^3^. The stress–strain curves of the PVA/PSBMA-PA hydrogel under 100% strain over eight cycles are depicted in Figure 2f,g, along with the maximum tensile strength and hysteretic energy for the respective cycles. The maximum tensile strengths of these eight cycles were approximately the same. However, a noticeable difference existed between the stretching and recovery curves in the first cycle, with a corresponding hysteretic energy of about 260 MJ/m^3^. This energy significantly decreased from the second to the eighth cycles, during which the stretching–recovery curves largely overlapped. This phenomenon could be attributed to the fact that some of the non-reversible bonds within the PVA/PSBMA-PA hydrogel were damaged during the first cycle of stretching and could not be recovered within a short period. Then, the tensile curves under different strain cycles were investigated, as shown in Figure 2h. It can be seen that the hysteresis energy of the hydrogel increased significantly with the increase of tensile deformation. Figure 2i–k correspond to the compression properties of PVA/PSBMA-PA hydrogel, where it can be seen that with the increase in PA content, the maximum compression strength of the hydrogel gradually decreased, and the maximum compression deformation gradually increased, similar to the tensile deformation. This was due to the existence of a hydrogen bonding interaction between PA and PVA, reducing the crosslinking within the PVA caused by the addition of 34% of PA. The compression and cyclic properties of the hydrogel during compression and deformation were investigated, as shown in Figure 2i. The cycling performance during deformation was investigated, as shown in Figure 2j,k. The compression cycling curves corresponding to different cycle times coincided, indicating consistent elasticity at 60% compressive strain. The maximum compressive strength and hysteresis energy remained nearly unchanged, demonstrating that the PVA/PSBMA-PA hydrogel possessed good deformation recovery and a certain degree of mechanical stability.

### 3.3. Electrical Properties of PVA/PSBMA-PA Hydrogel

**Figure 3:**
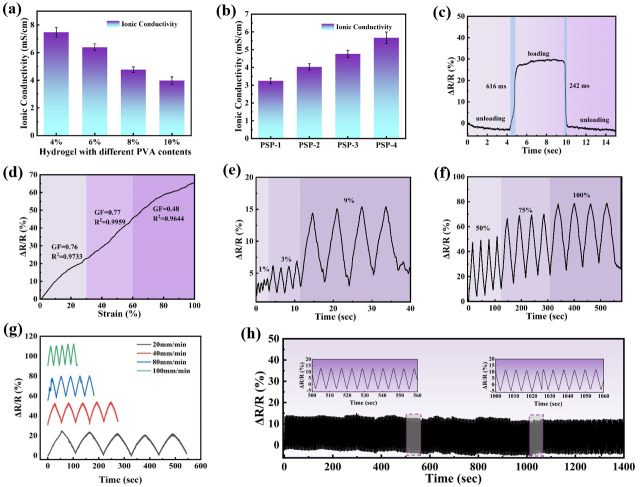
Electrical properties of hydrogels: (**a**) Hydrogel conductivity with different PVA contents and (**b**) hydrogel conductivity with different PA additions. (**c**) Response time and recovery time of hydrogels. (**d**) Relative resistance change of the PSP-3 hydrogel sensor, (**e**) change of the relative resistance under small deformation, (**f**) change of the relative resistance under large deformation, (**g**) change of the relative resistance of the hydrogel sensor at 30% deformation under different deformation frequencies, and (**h**) relative resistance change at 900 cycles for 20% deformation of the hydrogel sensor (zoomed-in graphs are localized zooms at 500~560 s and 1000~1060 s).

The inherent structural tunability of hydrogels enables compositional adjustments to customize their functional properties [49,50]. In this study, the conductive properties of hydrogels were realized by adding PA and SBMA. Since PVA itself is hardly conductive, the addition of the amphoteric ion SBMA enabled the conductivity of the hydrogel to be realized as 0.47 mS/cm (Figure S6), and on this basis, the conductivity of the PVA/PSBMA-PA hydrogel obtained by adding PA could be enhanced by an order of magnitude. Figure 3a,b mainly discuss the conductivity of hydrogel with different PVA contents and PA additions. With increasing PVA contents, the percentage of PSBMA in the PVA/PSBMA-PA hydrogel decreased, leading to a reduction in the conductivity. However, as the amount of PA increased, the conductivity was effectively enhanced to 5.8 mS/cm. Due to the excellent flexibility and conductivity of PVA/PSBMA-PA hydrogel, it was possible to realize the conductive properties of hydrogel when it was stretched. When the hydrogel was stretched, its length increased and its resistivity rose; conversely, when released, the resistivity decreased. As shown in Figure 3c, the PVA/PSBMA-PA hydrogel was utilized as a force-sensitive sensor, and the response time (616 ms) and recovery time (242 ms) of the loading–unloading process were investigated, which were found to be similar to those of general sensors [51]. The GF (gauge factor) of a hydrogel sensor is a critical metric for evaluating its strain sensitivity, which is defined as the material’s responsiveness to alterations in electrical resistance during mechanical deformation. A higher GF indicates a greater degree of discrimination capability by the sensor against minor strains. This calculation is outlined in Equation (S4) in the Supplementary Materials and is exclusively associated with the material’s properties, independent of its dimensions. As illustrated in Figure 3d, the parameter was partitioned into three distinct stages. The calculated values were 0.76, 0.77, and 0.48, respectively, in the strain ranges of 0–30%, 30–60%, and 60–100%. The GF of compressed hydrogels was also tested, as different loading methods resulted in different structural and conductive pathway changes in the hydrogels. Figure S7 shows the strain coefficients of the hydrogel at different compression amounts, and the strain coefficients gradually decreased as the deformation amount increased. As shown in Figure 3e,f, the curves of relative resistance versus deformation time for PVS/PSBMA-PA hydrogel sensors in the small and large strain ranges demonstrated the sensors’ capacity to detect strains as low as 1%, and they showed a clear and stable signal under different strains. The findings indicate that the PVA/PSBMA-PA hydrogel sensor possesses the capacity to monitor loads and systematically generate real-time feedback electrical signals. In addition, the hydrogel sensors showed a frequency-independent type, where the relative resistance remained stable at different deformation rates (Figure 3g). The relationship between the relative resistance of the hydrogel at deformation and different change frequencies was investigated (Figure 3h). When the hydrogel sensor was deformed by 20%, the relative resistance of the hydrogel at different deformation frequencies was basically the same, indicating no frequency dependence. The hydrogel sensor was exposed to 900 cycles of stretching, with each cycle involving a strain of 20%, and combined with the amplified relative resistance changes in the ranges of 500~560 s and 1000~1060 s, preliminary observations indicated that the hydrogel sensor exhibited adequate stability and did not demonstrate obvious signs of attenuation.

### 3.4. Anti-Swelling Properties

**Figure 4:**
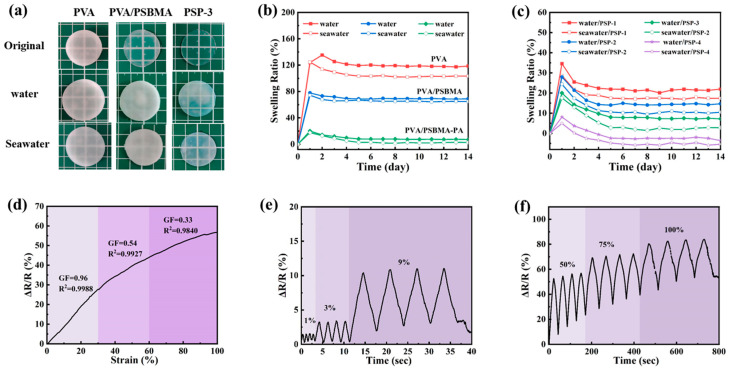
Swelling properties of hydrogels: (**a**) Photo of before and after swelling—hydrogels’ size change. (**b**) Swelling kinetics curves of different hydrogels. (**c**) Equilibrium swelling rate of hydrogels. (**d**) Relative resistance change of PSP-3 hydrogel after 14 days of swelling. (**e**) Change in relative resistance under small deformation. (**f**) Change in relative resistance under large deformation.

Anti-swelling properties of hydrogels are of particular significance in underwater environments [52,53]. Therefore, the swelling of PVA/PSBMA-PA hydrogel in solution (water and artificial seawater) was investigated. The swelling of the hydrogels was measured by detecting the weight change, and Figure 4a shows before and after photos comparing the swelling of PVA, PVA/PSBMA, and PSP-3 hydrogels in deionized water and artificial seawater. Figure 4b shows the swelling kinetics curves of different specimens immersed in different contexts for 14 days. It was obvious that the PVA/PSBMA hydrogel had a higher crosslinking density comparing with the pure PVA hydrogel, which was due to its internal double-network structure, and the swelling rate in both deionized water and artificial seawater was lower. The addition of PA on top of this further increased the electrostatic interaction and hydrogen bonding inside the PVA/PSBMA-PA hydrogel, which resulted in a lower swelling rate compared with that of the PVA/PSBMA hydrogel. The contact angle of the hydrogel was tested, as shown in Figure S4, and it was found that with the addition of PSBMA and PA, the contact angle increased, and the hydrophilicity did decrease. As illustrated in Figure 4c, the study examined the effect of PA incorporation on the swelling rate of the PVA/PSBMA-PA hydrogel. The findings demonstrated that with increasing PA addition, the swelling rate of the hydrogel decreased, and the lowest could reach 1.8% (artificial seawater), much better than general hydrogel [20,54,55]. In general, the swelling rate of hydrogels is negatively correlated with the crosslink density [56]. As the PA addition increased, the swelling rate decreased, which coincides with the increase in crosslink density in the previous section. In contrast to the other hydrogels in Table S1, the PSP hydrogel combines excellent anti-swelling properties with mechanical properties. Combined with Figure 4b,c, it was found that the hydrogel could reach the swelling equilibrium by immersing in the solution for about 2–3 days. Consequently, the following PVA/PSBMA-PA hydrogel sensors were evaluated for their underwater sensing capability following a three-day immersion period. Figure 4d shows the strain coefficient of the PVA/PSBMA-PA hydrogel after swelling, which was calculated to be 0.96 within 0–30%, 0.54 within 30–60%, and 0.33 within 60–100%. It changed compared to before swelling, due to the decrease in resistivity of the hydrogel after immersing in the solution [41]. Figure 4e,f investigate the change of the relative resistance with strain after the hydrogel swelling. It can be found that the hydrogel was still able to produce clear and distinct electrical signals for the corresponding strain with stability after swelling.

### 3.5. Human Motion Sensing of PVA/PSBMA-PA Hydrogel

**Figure 5:**
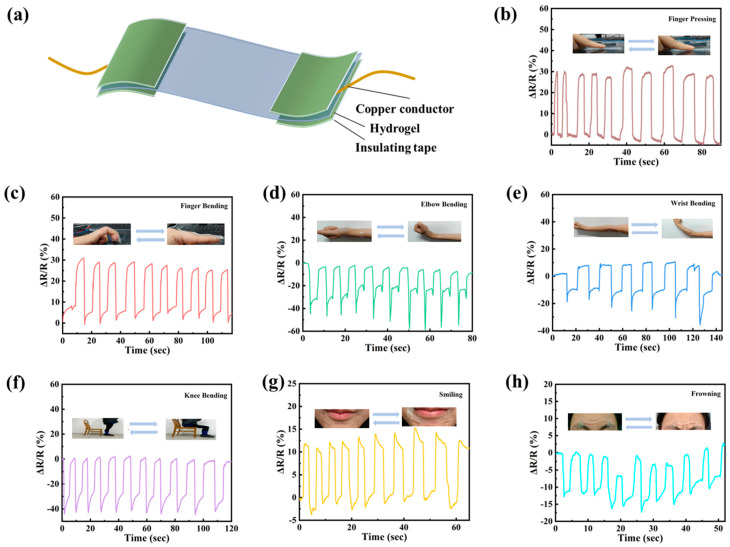
Motion sensing with hydrogel sensor. (**a**) Schematic of sensor assembly. Changes of relative resistance with different joint motions: finger pressing (**b**), finger bending (**c**), wrist bending (**d**), elbow bending (**e**), knee bending (**f**), smiling (**g**), and frowning (**h**).

Through rigorous testing of the hydrogel’s mechanical properties, it has been demonstrated that the material exhibited a maximum tensile strength of 304 kPa, with an approximate deformation capacity of 830%. The hydrogel has been engineered to function as a sensor, demonstrating its capacity to withstand testing of human movement. As illustrated in Figure 5a, the hydrogel sensor was composed of a hydrogel and affixed to the skin to be analyzed. The hydrogel was encapsulated at both ends with copper and waterproof tapes, and lead wires were connected to an electrochemical workstation to collect the electrical signals. As demonstrated in Figure 5b, the electrochemical signals corresponding to the behavior of finger compression were initially displayed. At the same time, the hydrogel sensor could also obtain relative resistance change curves based on different degrees of finger bending (Figure S6). Figure 5c–f show that the hydrogel sensor detected the corresponding actions of finger bending, elbow bending, wrist bending, and knee bending. These terms of signals were used to denote the act of flexing the joints of the upper limbs. As evidenced by the figures presented, the movement of disparate components can be readily discerned based on the waveform of the curves. Furthermore, it was ascertained that the execution of the identical action resulted in the generation of signals that were nearly indistinguishable, thereby substantiating the reliability and repeatability of the gel sensor. In addition, the use of hydrogel-based sensors has the potential to facilitate the detection of human micro-expression, as shown in Figure 5g,h, where the hydrogel sensor was affixed to the volunteer’s chin and forehead, and by detecting the muscle changes in the corresponding areas, the micro-expressions of the person can be distinguished (“smile” and “frown”).

### 3.6. Underwater Motion Sensing of PVA/PSBMA-PA Hydrogel

**Figure 6:**
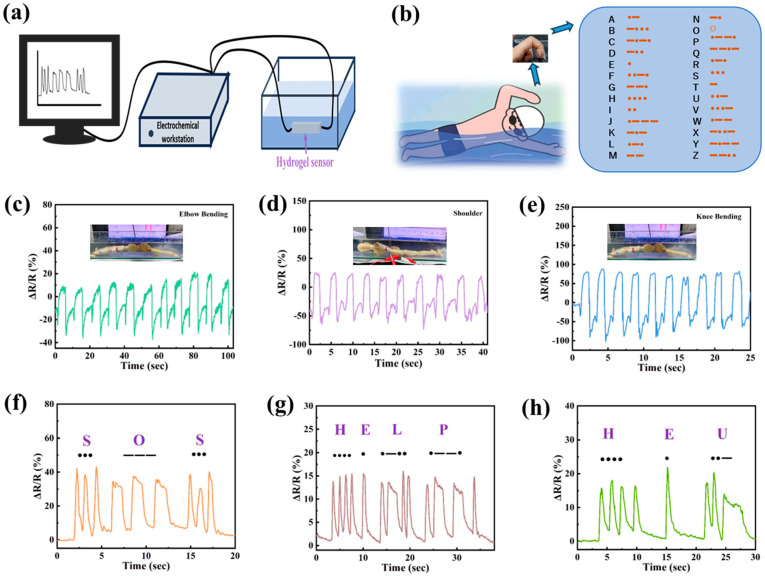
PVA/PSBMA-PA hydrogel underwater sensing to detect motion and signal transmission: (**a**) Schematic of hydrogel sensor connection. (**b**) Schematic diagram of the signal transmission principle of the hydrogel sensor with a comparison table of the translation of Morse code. (**c**) Elbow, (**d**) Shoulder, and (**e**) Knees signal transmission. Signal transmission: (**f**) “SOS” signal, (**g**) “HELP” signal, and (**h**) “HEU” signal.

The results of the solubility test indicated that the water-soluble rates of the water-soluble and artificial gels were 7.2% and 1.8%, respectively. These results suggest that the gels have excellent solubility control, making them suitable for use in hydrodynamic movement analysis. Figure 6a illustrates the circuit diagram of a hydrogel sensor connected underwater. The experiment was conducted using dolls in place of human subjects, with hydrogels attached to various joints of the dolls (Figure 6c—elbow, Figure 6d—shoulder, and Figure 6e—knee). Due to the difference in the amplitude and type of movement of the different joints during the movement, the corresponding change in the relative resistance of the sensors will also be different [49]. As illustrated in Figure 6b, the principle of signal transmission in hydrogel sensors is based on the variation in “compression rebound” rates of the hydrogel sensor, which corresponds to different “dot” and “cross” signals in the internationally recognized Morse code. Specifically, the phenomenon of “rapid compression rebound” is analogous to the ‘dot’ signal in Morse code, while the process of “compression held for a period of time before rebound” corresponds to the ‘cross’ signal. The different “point” and “cross” signals were used to form the international common “Morse code” to realize the signal transmission of hydrogel underwater, as shown in Figure 6c–e, and by changing the different “compression return” signals, the signal transmission was realized. As modifying in Figure 6f–h, by modifying different combinations of “compression response” corresponding signals, the transmission of “SOS,” “HELP,” and “HEU” signals was successfully accomplished. In this paper, although underwater signal transmission can be realized through the assembled hydrogel, the simplification of wireless transmission has not yet been realized, and we hope to continue the exploration in this direction in the future.

## 4. Conclusions

In this paper, a hydrogel strain sensor was proposed by crosslinking PVA and PSBMA and introducing hydrogen bonding and electrostatic interaction by PA. Based on the existence of hydrogen bonding between PVA, PSBMA, and PA, as well as the electrostatic interaction, the SPVA/PSBMA-PA hydrogel exhibited excellent resistance to swelling. The equilibrium swelling rates of the hydrogels were only 7.2% and 1.8% after 14 days of immersion in deionized water and artificial seawater, respectively. Meanwhile, the PVA/PSBMA-PA hydrogel showed high strength (304 kPa), high conductivity (5.8 mS/cm), and a strain coefficient of 0.77. The PVA/PSBMA-PA hydrogel was fabricated into a sensor, and after 900 cycles at 20% strain, the hydrogel sensor showed no significant signal degradation. In addition, the hydrogel sensor was able to quickly and accurately output clear and stable signals against the target motion in both terrestrial and underwater environments. Therefore, the prepared swelling-resistant hydrogel sensor presents a new idea for wearable electronic devices in the underwater environment.

## Data Availability

Data are contained within the article and Supplementary Materials.

## Supplementary Materials

The following supporting information can be downloaded at: https://www.mdpi.com/article/10.3390/nano15131027/s1, Figure S1. Energy dispersive spectroscopy (EDS) elemental mapping of the PVA/PSBMA-PA hydrogel. Figure S2. FTIR spectra of SBMA, the PVA hydrogel, PVA/PSBMA hydrogel, and PVA/PSBMA-PA hydrogel. Figure S3. SEM images of (a) PVA hydrogel; (b) PVA/PSBMA hydrogel; (c) PVA/PSBMA-PA. Figure S4. Water contact angle of (a) PVA hydrogel; (b)PVA/PSBMA hydrogel; (c) PVA/PSBMA-PA hydrogel. Figure S5. Stress-strain curves of different hydrogels. Figure S6. Ionic conductivity of PVA/PSBMA hydrogel and PVA/PSBMA-PA hydrogel. Figure S7. Relative resistance changes of the PSP-3 hydrogel sensor as a function of the applied compressive strain (0–60%). Figure S8. Relative resistance changes of the PSP-3 hydrogel sensor with different finger bend angles. Table S1. Properties of PVA/PSBMA-PA hydrogel compared with previous literature reports. Refs. [24,56,57,58,59,60,61,62,63] are cited in the supplementary materials.
