# Supplementary material for: High Anti-Swelling Zwitterion-Based Hydrogel with Merit Stretchability and Conductivity for Motion Detection and Information Transmission
Source: Nanomaterials (Basel). 2025 Jul 2;15(13):1027. doi: 10.3390/nano15131027 (PMC12250818; doi:10.3390/nano15131027)
Supplement: Supplementary file 1 [file nanomaterials-15-01027-s001.zip › nanomaterials-3692415-supplementary.pdf]

Supporting Information

High Anti-Swelling Zwitterion-Based Hydrogel with Merit Stretchability and Conductivity for Motion Detection and Information Transmission

Qingyun Zheng, Jingyuan Liu *, Rongrong Chen, Qi Liu, Jing Yu, Jiahui Zhu and Peili Liu

Characterization:

Attenuated total reflection infrared spectroscopy: The transmission and reflection of light from the samples in the region of 4000-700 cm^{-1} was recorded using an ALPHA II Fourier transform infrared spectrometer (Bruker, Germany) to obtain the positions of the absorption peaks of the different vibrational modes of the compound molecules and the infrared spectra. This process was performed to achieve the purpose of qualitative and semi-quantitative analysis of the functional groups on the surface of the materials.

Scanning electron microscope and energy dispersive spectroscopy: To characterize the material morphology, a scanning electron microscope (APREO S LOVAC) from Thermo Fisher Scientific was utilized. The specific operation was as follows: after vacuum freeze-drying of the hydrogel, it was adhered to the conductive adhesive, sprayed with gold to improve its conductivity, and then observed at a voltage of 10.0 kV and 5 kV.

Mechanical properties**:** The mechanical properties of the hydrogels were tested using a universal testing machine (Instron 3365). For the tensile test, the samples were cut into a dumbbell shape with customized mold dimensions (10 mm (length) × 25 mm (width) × 2 mm (thickness)) and tested at a speed of 50 mm/min. In the compression test, the size of the sample was a cylinder with a height of 10 mm and a diameter of 3 mm. The elastic modulus of hydrogels was determined from the slope of the stress-strain curves. Toughness was determined based on the integrated area of tensile curves.

Swelling properties**:** Hydrogels were weighed and immersed in various solvents at room temperature. Then their weights were measured once a day. The swelling ratio

(SR) was calculated according to the following equation (S1):

$$SR = \frac{W_s}{W_0} \times 100\% - 1 \qquad (S1)$$

Where W_0 is the weight of the original hydrogel and W_S is the weight of the swollen hydrogel.

***Conductivity properties and sensing properties*:** The electrical property of the hydrogel was evaluated by the electrochemical workstation (Chi660E, CH Instruments Ins, China). The resistance of the columnar hydrogels was recorded by Amperometric Current (i-t), and the ionic conductivity (σ, S m^{-1}) was obtained according to the following equation (S2):

$$\sigma = \frac{L}{R \times S} \qquad (S2)$$

Where R is the resistance value (Ω), L and S are the height (m) and the sectional area of the hydrogel (m^2), respectively. The sensing properties of the hydrogel were evaluated by a universal testing machine (Instron 3365) and an electrochemical workstation (Chi660E, CH Instruments Ins, China). The hydrogel sensor was fixed on the platform and connected to the electrochemical workstation with a wire, and then different pressure was applied to the sensor to stimulate the signal, and the current change in the process was recorded in real time. The real-time resistance of the hydrogel was derived from i–t curve, and the relative resistance change was determined by the following equation (S3):

$$\frac{\Delta R}{R_0} = \frac{R - R_0}{R_0} \times 100\% \qquad (S3)$$

The sensitivity (GF) is the slope of the linear fitting curve between the relative resistance change of the strain sensor and the strain variable, reflecting the sensitivity of the sensor to strain. It was defined as the following equation (S4):

$$GF = \frac{(R-R_0)/R_0}{\varepsilon} \qquad (S4)$$

Motion monitoring: Tested with an electrochemical workstation (Chi660E, CH

Instruments Ins, China), the assembled hydrogel sensors were strapped to the volunteer’s joints (elbow, knee, shoulder, etc.) to simulate the human body's underwater motion through the mannequins. By defining different “compression return” rates corresponding to different “dot” and “cross” signals, “compression returns immediately” corresponds to “dot” and “return after compression and holding time” is defined as “cross”. “Dot” and ‘cross’ signals are used to form the internationally recognized ‘Morse code’, which realizes the signal transmission of hydrogel underwater.

Supplementary Figures

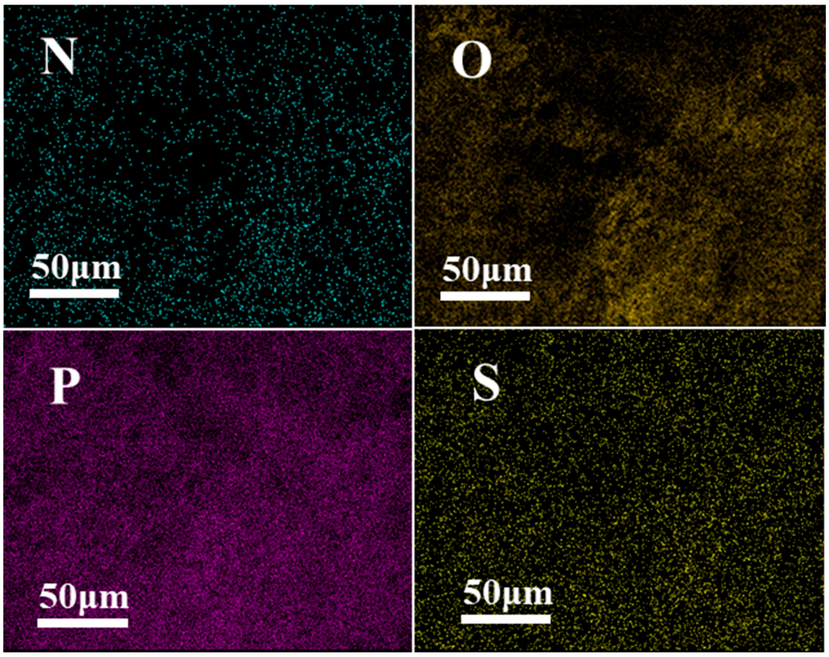

Figure S1. Energy dispersive spectroscopy (EDS) elemental mapping of the PVA/PSBMA-PA hydrogel.

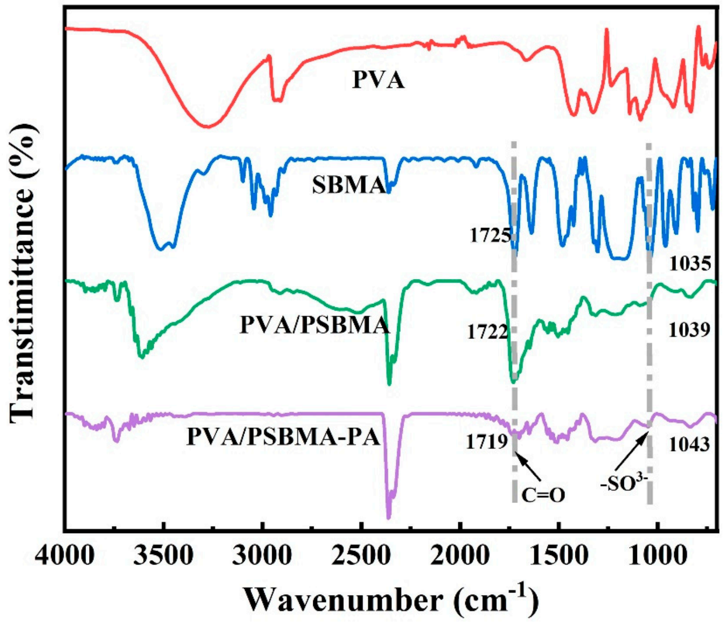

Figure S2. FTIR spectra of SBMA, the PVA hydrogel, PVA/PSBMA hydrogel, and PVA/PSBMA-PA hydrogel.

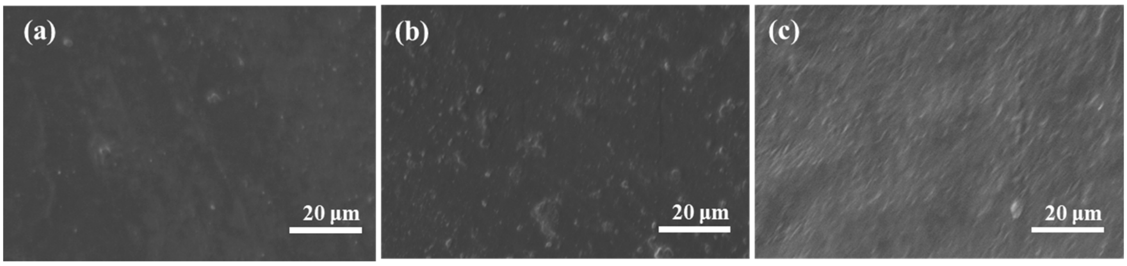

Figure S3. SEM images of (a) PVA hydrogel; (b) PVA/PSBMA hydrogel; (c) PVA/PSBMA-PA hydrogel.

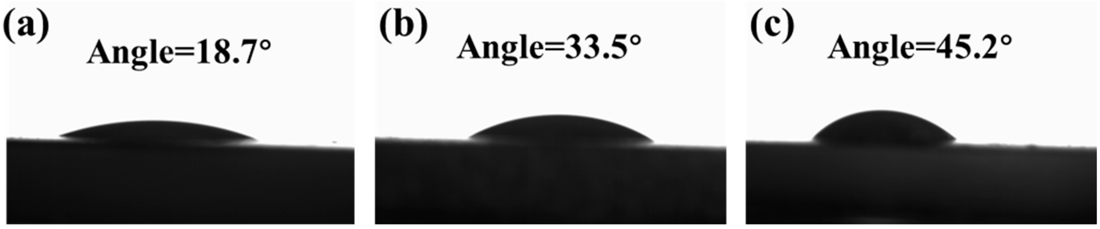

Figure S4. Water contact angle of (a) PVA hydrogel; (b)PVA/PSBMA hydrogel; (c) PVA/PSBMA-PA hydrogel.

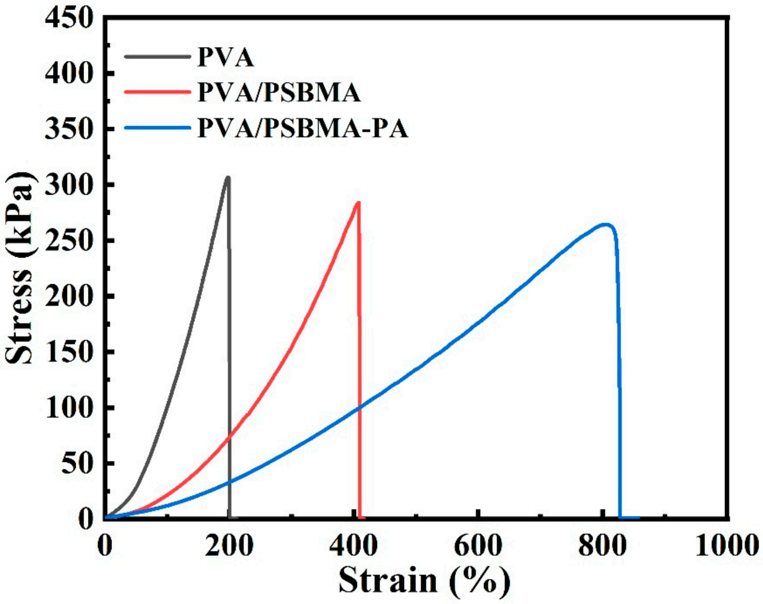

Figure S5. Stress-strain curves of different hydrogels.

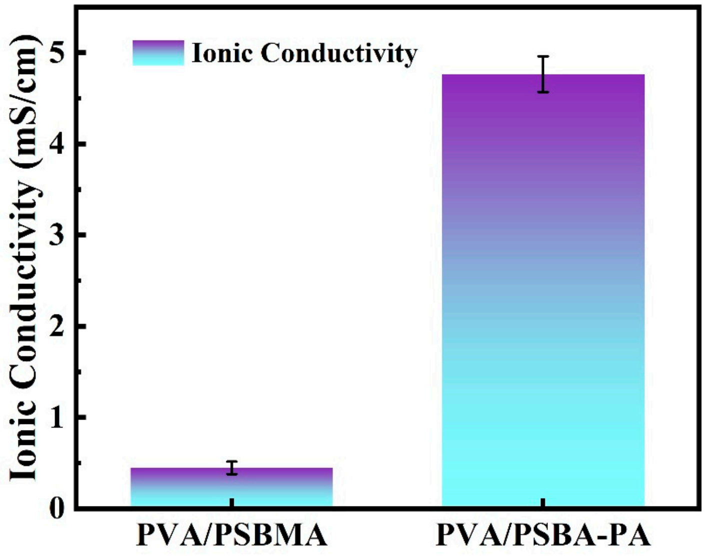

Figure S6. Ionic conductivity of PVA/PSBMA hydrogel and PVA/PSBMA-PA hydrogel.

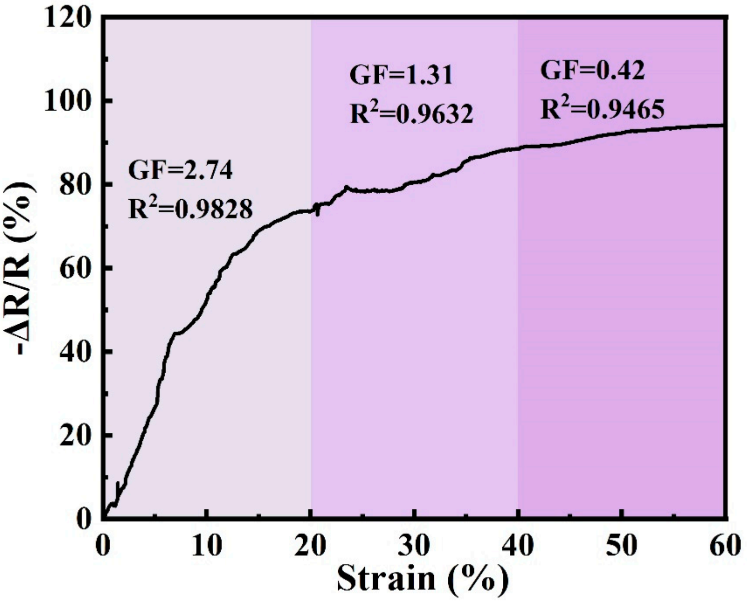

Figure S7. Relative resistance changes of the PSP-3 hydrogel sensor as a function of the applied compressive strain (0 – 60%).

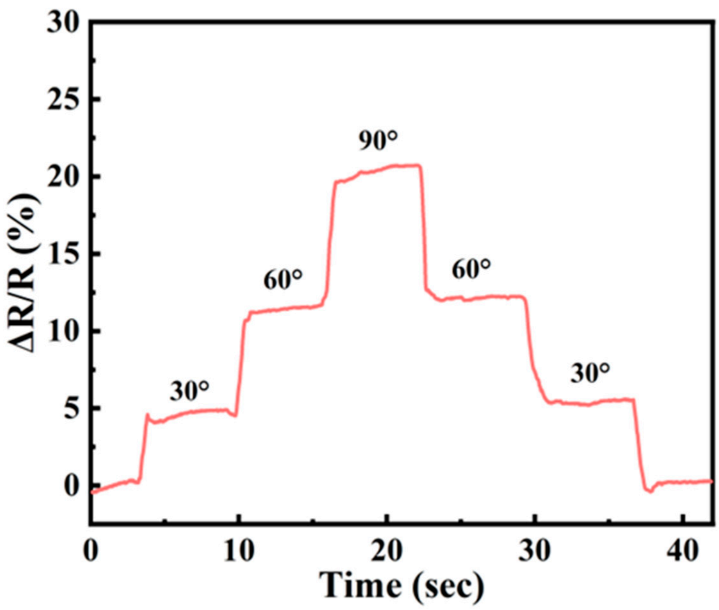

Figure S8. Relative resistance changes of the PSP-3 hydrogel sensor with different finger bend angles.

Table S1. Properties of PVA/PSBMA-PA hydrogel compared with previous literature reports.

Materials	Ultimate stress (kPa)	Tensile length (%)	Tensile Gauge factor (GF)	Swelling ratio in water (%)	Swelling ratio in seawater (%)	References
PVA /HPMC/PA/SBMA/$ZnCl_2$[1]	2100	750	1.1	0.1 (150h)	21%(150h)	F Wang et al.,2025
γ-PGA/PVA/PEDOT:PSS/TA[2]	897	1495.5	4.33	13(100h)	-	C.X. Gao et al.,2023
PMAm/ LSA[3]	113	183	2.1	3(7day)		R.L. Zhang et al., 2025
A_xH_y ionic glycerol-hydrogel[4]	127	580	1.73	17(500min)	-	M.L. Guo et al., 2024
Janus TA@PVA/PSBMA[5]	198	320	1.56	23.2 (14day)	-	L.D. Xu et al.,2024
PSBMA/PVA/Borax [6]	305	300	-	3.34 (35h)	0.97(35h)	H.Y. Liu et al., 2021
HEMA/ PVP/ PPy[7]	557.7	451.4	1.31	21.1(120h)	21(120h)	K. J. Zhong et al., 2025
Acr-PVA-g-PSBMA[8]	40	25	-	5.5 (4day)	-	X. Yang et al., 2020
P（AA-co-LMA）-CTAB[9]	1600	950	0.42	6(15day)	-	C.Y. Qi et al., 2022
PVA/PSBMA-PA	304	830	0.77	7.2(14day)	1.8(14day)	this work

Reference

[1] F. Wang, H.Q. Zhang, C.L. Liu, W. Bao, Y.J. Hu, and X. Maimaitiyiming, High toughness, high stability and low hysteresis PVA /HPMC/PA/SBMA/ ZnCl2 conductive hydrogels for wearable flexible electronics for multifunctional sensors and supercapacitors, Carbohydrate Polymers, 361, (2025), 123644.

[2] C.X. Gao, D.Y. Zheng, B.C. Long, Z.W. Chen, J.D. Zhu, and Q. Gao, Anti-swelling and adhesive γ-PGA/PVA/PEDOT:PSS/TA composite conductive hydrogels for underwater wearable sensors, European Polymer Journal, 201, (2023), 122590.

[3] R.L. Zhang, L.R. Liu, Z. Ge, H.Y. Ren, W.T. Wang, M.Y. Zhu, and C.H. Luo, Coordination hydrogel enables an extremely stable, highly sensitive, self-healable and robust underwater sensor, European Polymer Journal, 233, (2025), 114010.

[4] M.L. Guo, Y. Zheng, Y.Q. Feng, W.H. Yang, J.J. Pu, T. Ren, S.C. Hu, and Y.P. Wu, Anti-freezing and conductive hydrogel with water-resistant properties for flexible underwater sensors, Colloids and Surfaces a-Physicochemical and Engineering Aspects, 702, (2024), 135000.

[5] L.D. Xu, Y.M. Wang, H. Li, Z.Q. Hou, X. Miao, G. Miao, F.C. Li, J.W. Lu, G.A. Ren, and X.T. Zhu, Janus Hydrogel with Both Sticky Adhesion and Slippery Antifouling Properties for Strain Sensing, Acs Applied Polymer Materials, 6, (2024), 2339-2348.

[6] H.Y. Liu, L. Yang, B.J. Dou, J.W. Lan, J.J. Shang, and S.J. Lin, Zwitterionic hydrogel-coated cotton fabrics with underwater superoleophobic, self-healing and anti-fouling performances for oil-water separation, Separation and Purification Technology, 279, (2021), 119789.

[7] K.J. Zhong, J. Xiang, Y. Liu, C.L. Song, Y.T. Mu, T. Yao, K. Zhao, T.F. Gu, P.X. Jia, and W.Y. Zhang, Fabrication of a high strength, swelling resistance, and conductive hydrogel for flexible underwater sensor via ketalization, Chemical Engineering Journal, 511, (2025), 161885.

[8] X. Yang, D. Sha, L. Sun, L. Chen, J.D. Xu, K. Shi, C. Yu, B.L. Wang, and X.L. Ji, Charged group-modified poly(vinyl alcohol) hydrogels: Preparation and antibacterial property, Reactive & Functional Polymers, 154, (2020), 104635.

[9] C.Y. Qi, Z.X. Dong, Y.K. Huang, J.B. Xu, and C.H. Lei, Tough, Anti-Swelling Supramolecular Hydrogels Mediated by Surfactant-Polymer Interactions for Underwater Sensors, ACS Applied Materials & Interfaces, 14, (2022), 30385-30397.
